# Anticancer and Anti-Inflammatory Properties of Chitin and Chitosan Oligosaccharides

**DOI:** 10.3390/jfb6010033

**Published:** 2015-01-14

**Authors:** Kazuo Azuma, Tomohiro Osaki, Saburo Minami, Yoshiharu Okamoto

**Affiliations:** Department of Veterinary Clinical Medicine, School of Veterinary Medicine, Tottori University, 4-101 Koyama-minami, Tottori 680-8553, Japan; E-Mails: tosaki@muses.tottori-u.ac.jp (T.O.); saburominami@ncn-t.net (S.M.); yokamoto@muses.tottori-u.ac.jp (Y.O.)

**Keywords:** oligomers, glucosamine, *N*-acetyl-d-glucosamine, chitin, chitosan, cancer, anti-inflammatory, inflammatory bowel disease

## Abstract

Previous reports indicate that *N*-acetyl-d-glucosamine oligomers (chitin oligosaccharide; NACOS) and d-glucosamine oligomers (chitosan oligosaccharide; COS) have various biological activities, especially against cancer and inflammation. In this review, we have summarized the findings of previous investigations that have focused on anticancer or anti-inflammatory properties of NACOS and COS. Moreover, we have introduced recent evaluation of NACOS and COS as functional foods against cancer and inflammatory disease.

## 1. Introduction

Chitin (β-(1-4)-poly-*N*-acetyl-d-glucosamine) is widely distributed in nature and is the second most abundant polysaccharide after cellulose 1 [1]. Chitin occurs as the major structural component in the exoskeleton of crab and shrimp shells and the cell wall of fungi and yeast [2]. As chitin is not readily dissolved in common solvents, it is often converted to its more deacetylated derivative, chitosan [3,4,5]. Even though chitin and chitosan are known to have important functional activities, their poor solubility makes them difficult to use in food and biomedical applications [6]. In contrast, the hydrolyzed products of chitosan—*N*-acetyl-d-glucosamine oligomers (chitin oligosaccharide; NACOS) and d-glucosamine oligomers (chitosan oligosaccharide; COS) are readily soluble in water because of their shorter chain lengths [7]. The low viscosity and greater solubility of COS at neutral pH have attracted the interest of many researchers to utilize chitosan in its oligosaccharide form. NACOS and COS are generated by depolymerization of chitin or chitosan by using acid hydrolysis, hydrolysis by physical methods, and enzymatic degradation [8].

Recently, many reports have indicated that NACOS and COS possess numerous biological activities. However, most of these studies were performed either *in vitro* or via intravenous (i.v.) or intraperitoneal (i.p.) administration. More recently, the anticancer and anti-inflammatory effects of orally administered NACOS or COS have been described. In this review, we focus on these properties of NACOS and COS by first summarizing the findings of previous studies and then discussing the potency of NACOS and COS as functional foods against cancer and inflammation.

## 2. NACOS, COS, and Their Derivatives as Anti-Cancer Agents

### 2.1. Anti-Cancer Activities of NACOS and COS

Nam *et al.* reported chemo-preventive effects of COS in colon cancer cells [9]. The effects were evaluated by measuring the activities of enzymes quinine reductase (QR), ornithine decarboxylase (ODC), and glutathione-S-transferase (GST) as well as glutathione (GSH) levels and cyclooxygenase-2 (COX-2) expression in human colorectal adenocarcinoma cell line, HT-29, treated with COS. These results indicate that COS exerts its chemopreventive effect against colon cancer by increasing QR and GST activities and GSH levels and by inhibiting ODC activity and COX-2 expression *in vitro*. In another study, Nam *et al.* also showed that COS pretreatment inhibited pro-inflammatory cytokine-mediated nitric oxide (NO) production, inducible NO synthase (iNOS) expression, and invasiveness of HT-29 cells [10]. Quan *et al.* have discovered COS to have antiangiogenic activity through an unclear mechanism but hypothesized it to be via inhibition of heparanase [11]. They have also shown that MDA-MB-231 cells treated with COS had a concentration-dependent reduction in matrix metalloproteinase-9 (MMP-9) secretion and activity as well as inhibition of their invasiveness through a matrigel-coated membrane [12].

Shen *et al.* have investigated the antitumor and antimetastatic potential as well as pathways affected by COS extracted from fungi, in human hepatocellular carcinoma cell line, HepG2 [13]. They discovered that *in vitro* COS significantly inhibited cell proliferation, reduced the percentage of cells in S-phase, and decreased the rate of DNA synthesis in the cells. Further analysis of expression of cell cycle-related genes revealed that p21 was upregulated, while proliferating cell nuclear antigen (PCNA), cyclin A, and cyclin dependent kinase (CDK)-2 were downregulated. Moreover, they observed that MMP-9, an enzyme associated with metastasis, could be inhibited by COS in Lewis lung carcinoma (LLC) cells. During animal studies, they discovered that intraperitoneal injections of COS inhibited the growth of HepG2 xenografts in severe combined immune deficient (SCID) mice. Furthermore, in an LLC mouse model of primary tumor and metastasis, COS administration was found to inhibit tumor growth, decrease the number of metastatic colonies in lung, and prolong the survival time of the animals.

It has been postulated that the tumor inhibitory effects of NACOS and COS are potentially related to their ability to induce lymphocyte cytokines thorough increased T-cell proliferation. Essentially, the antitumor mechanisms of NACOS and COS are presumably enhanced by acquired immunity via acceleration of T-cell differentiation, which in turn increases cytotoxicity and maintains T-cell activity [14]. Park *et al.* have examined the effects of molecular weight and degree of deacetylation of chitosan oligosaccharides on their antitumor activity [15]. They fractionated chitosan oligosaccharide (CTS-OS) by gel-filtration chromatography into two major fractions: (1) COS, consisting of glucosamine (GlcN)(*n*), *n* = 3–5, with a 100% degree of deacetylation (DDA) and (2) COS, consisting of (GlcN)(5) as the minimum residues and varying number of *N*-acetylglucosamine (GlcNAc)(*n*), *n* = 1–2, with DDA about 87.5% in random order. The cytotoxic potential of these, expressed as EC(50) (the concentration needed for 50% cell death), of CTS-OS, COS, and HOS against cancer cell lines—PC3 (human prostate), A549 (human lung), and HepG2 (human hepatoma), was determined to be 25 μg/mL, 25 μg/mL, and 50 μg/mL, respectively. The high molecular weight chitosan (HMWC) was approximately 50% less effective as compared to both CTS-OS and COS. This data indicate that the molecular weight and DDA of chitosan oligosaccharides are important factors for suppressing cancer cell growth. Table 1 is a summary of the literature on these studies.

**Table 1:** A summary of anti-cancer activities of NACOS, COS and its derivatives.

Preparation	Cells or Model	Major Results	Ref.
COS	HT-29 (*in vitro*)	Increased QR and GST activities and GSH levels; Inhibited ODC activities and COX-2 expression	[9]
COS	HT-29 (*in vitro*)	Inhibited NO production and iNOS expression	[10]
COS	MDA-MB-231 (*in vitro*)	Reduced MMP-9 secretion and activities	[12]
COS	HepG2 (*in vitro*)	Reduced cells in S-phase and decreased the rate of DNA synthesis; Upregulated p21 and downregulated PCNA, cyclin A and CDK-2	[13]
COS	LLC (*in vitro*)	Inhibited MMP-9	[13]
COS	HepG2 (*in vivo*)	Inhibited the tumor growth	[13]
COS	LCC (*in vivo*)	Inhibited the tumor growth and decreased the number of metastatic colonies	[13]
NACOS, COS	Meth-A (*in vivo*)	Enhanced acquired immunity	[14]
COS	PC-3, A549 (*in vitro*)	Suppressed cancer cell growth	[15]
CSO-SA	MCF-7, A549, Bel-7402 (*in vitro*)	Discovered anti-cancer activities of podophyllotoxin loaded on CSO-SA micelles	[16]
CSOSA-g-PEI	Hala, MCF-7 (*in vitro*)	CSOSA-g-PEI/plasmid suppressed tumor growth	[17]
CSOAA	FaDu (*in vitro*)	Showed cytotoxicity. DOX-loaded CSOAA-based nanoparticle was highly uptake	[18]
Gal-CSO/ATP	HepG2 (*in vitro*)	Gal-CSO/ATP nanoparticle showed high cytotoxicity	[19]
FA-PEG-COL	OVK18 #2 (*in vitro*)	FA-PEG-COL nanoparticles accumulated in tumors	[20]
FcCOS	–	The release of drug was enhanced in the oxidative condition and low pH	[21]

### 2.2. Anti-Cancer Activities of COS Derivatives

The utility of COS derivatives in targeted drug delivery/gene therapy has also been extensively investigated. Huang *et al.* have studied the derivatives of stearic acid-g-chitosan oligosaccharide (CSO-SA) as potential carriers for intracellular delivery of anticancer agents [16]. They compared the cytotoxicity of podophyllotoxin (PPT) in a free state *vs.* PPT loaded on CSO-SA micelles (CSO-SA/PPT) against human cancer cell lines, breast carcinoma (MCF-7), lung cancer (A549), and hepatoma (Bel-7402) and discovered better anticancer activity in the micelle-loaded PPT. This higher cytotoxicity observed can be attributed to faster PPT transport into tumor cells mediated by CSO-SA micelles. Hu *et al.* have evaluated the low-molecular weight polyethylenimine-conjugated stearic acid-g-chitosan oligosaccharide (CSOSA-g-PEI) for gene delivery and therapy [17]. The designed CSOSA-g-PEI had notable ion-buffering property and DNA-binding capacity and could form positively-charged, nanosized particles (100–150 nm) with plasmid DNA, and *in vitro* gene transfection tests demonstrated that CSOSA-g-PEI presented much lower cytotoxicity than and a transfection efficiency comparable to Lipofectamine 2000 in human cancer cell lines, Hela and MCF-7. The transfection efficiency of CSOSA-g-PEI/pDNA could be further enhanced in the presence of serum or by adding arginine during incubation of CSOSA-g-PEI micelles with plasmid DNA. Further biodistribution experiments demonstrated that CSOSA-g-PEI conjugate are highly localized and are increasingly accumulated in the tumor tissue. Efficacy evaluation *in vivo* showed that CSOSA-g-PEI/plasmid pigment epithelium-derived factor administered intravenously could effectively suppress tumor growth (>60% tumor inhibition) without any systemic toxicity. Termsarasab *et al.* have tested chitosan oligosaccharide-arachidic acid (CSOAA) conjugate for the development of self-assembled nanoparticles intended for doxorubicin (DOX) delivery [18]. The DOX-loaded CSOAA-based nanoparticles were spherical in shape with mean diameter of 130 nm and were positively charged. Results of *in vitro* release test revealed that DOX-loaded CSOAA-based nanoparticles had a sustained and pH-dependent drug release profile. In addition, CSOAA showed negligible cytotoxicity in the human head and neck cancer cell line, FaDu and cellular uptake of DOX was higher in the nanoparticle-treated cells in comparison with free DOX-treated cells. Zhu *et al.* have evaluated the characteristics of galactosylated chitosan oligosaccharide (Gal-CSO) and adenosine triphosphate (ATP) (Gal-CSO/ATP) nanoparticles [19]. They estimated the cytotoxicity of Gal-CSO/ATP nanoparticles in HepG2 cells by using methyl tetrazolium (MTT) assay, and calculated the half maximal inhibitory concentration (IC_50_) values. Their results showed that the nanoparticles had low cytotoxicity but were taken up by HepG2 cells owing to expression of asialoglycoprotein receptor (ASGP-R) on their surface.

Li *et al.* have demonstrated targeted delivery of siRNA to the cancer site following conjugation with folic acid-poly (ethylene glycol)-chitosan oligosaccharide lactate (FA-PEG-COL) nanoparticles [20]. In this study, the efficiency of FA-PEG-COL nanoparticles in localizing in tumors was visualized in BALB/c mice bearing OVK18 #2 tumor xenograft by using *in vivo* imaging, and the researchers discovered that FA-PEG-COL nanoparticles accumulated substantially in tumors as compared to non-targeting COL nanoparticles. Xu *et al.* have reported a detailed investigation on the oxidation and pH response of ferrocene-modified chitosan oligosaccharide (FcCOS) nanoparticles for 5-fluorouracil (5-FU) delivery [21]. The dispersion of FcCOS nanoparticles depends strongly on pH change and in this study, the researchers showed that 5-FU, the model drug that was efficiently loaded in FcCOS nanoparticles (approximately 238 nm), was released more efficiently with decreasing pH under bubbled N_2_. Interestingly, the cumulative release of sample under bubbled air and pH of 3.8 was higher at 59.64%, while under bubbled N_2_ it was 49.02%. These results suggest a synergistic effect of oxidative conditions and low pH in enhancing the disassembly of FcCOS nanoparticles and the release of drug molecules. Table 1 is a summary of the literature on these studies.

## 3. Anti-Cancer Effects of NACOS and COS Following Oral Administration

**Figure 1:**
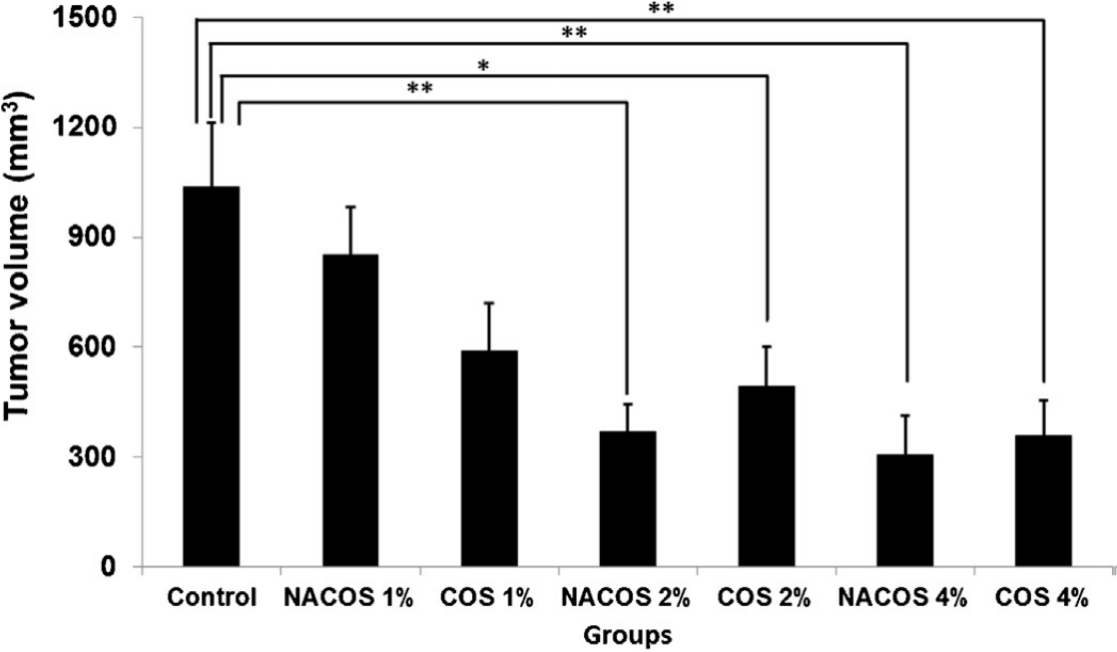
Effect of orally administered NACOS and COS on tumor growth. The effects of orally administered NACOS and COS were evaluated using colon 26 bearing mouse model. Mice were fed 1%, 2% or 4% NACOS or COS contained diet. Data represent the mean ± standard error. *n* = 8–10 in each groups. ** indicates *p* < 0.01 and * indicates *p* < 0.05 as compared to the control group (Tukey-Kramer test). Reprinted with permission. Copyright 2014 Elsevier [22].

In most animal studies that have evaluated the anticancer properties of NACOS and COS, the route of administration has been either i.v. or i.p., and there is not much reported on the beneficial effects of NACOS and COS following oral administration. We recently assessed the anticancer properties of orally administered NACOS and COS in a mouse model of colon cancer using the cell line, colon-26 [22]. We observed that in animals receiving either COS (2% and 4%) or NACOS (2% and 4%), tumor volumes were significantly lower than those in control group (*p* < 0.05) (Figure 1). Moreover, the active cell proliferation seen in control group was markedly suppressed in the NACOS and COS groups, and instead, necrotic cells were widely observed in the tumors in these animals. Serum levels of interleukin-12p70 and interferon-γ were also considerably increased in the NACOS and COS groups (*p* < 0.01, Table 2). Collectively, these results indicate that the anticancer effects of NACOS and COS following oral administration could be mediated by enhanced innate immunity. Previous reports have indicated that the inhibitory effect of COS on tumor growth was most likely related to its ability to induce lymphocyte cytokines by increasing T-cell proliferation. Mainly, adaptive immunity is thought to have enhanced the antitumor mechanism of COS by accelerating T-cell differentiation, which in turn increases cytotoxicity and maintains T-cell activity [14]. Studies have demonstrated that the antitumor effects of certain low molecular weight chitosans, such as water-soluble 21- or 46-kDa molecules that form low viscosity solutions, in mice bearing sarcoma (180 tumors) can be attributed to an increase in natural killer (NK) cell activity [23,24]. Another separate report stated that a low molecular weight, water-soluble chitosan and COS could prevent tumor growth by serving as immunomodulator in enhancing cytotoxic activity against tumors [25]. In certain cases of skin disease, low molecular weight, water-soluble chitosan and COS have been shown to activate macrophages via the production of cytokines, interferon (IFN)-γ and interleukin (IL)-12, in intraepithelial lymphocytes [26]. These observations strongly suggest that oral administration of NACOS and COS stimulates the production of IFN-γ and IL-12.

**Table 2:** Effect of orally administered NACOS and COS on serum cytokine levels. Reprinted with permission. Copyright 2014 Elsevier [22].

Cytokines	Control	NACOS 4%	COS 4%
IFN-γ (pg/mL)	0.6 ± 0.2	8.3 ± 0.5 **	8.4 ± 0.3 **
IL-12 (pg/mL)	11.2 ± 1.3	25.3 ± 3.5 **	23.5 ± 3.0 **
TNF-α (pg/mL)	13.9 ± 1.6	13.4 ± 1.3	12.7 ± 0.8

Mice were fed 1%, 2% or 4% NACOS or COS contained diet. Mean ± Standard error; *n* = 6 in each group; ** *p* < 0.01 as compared to the control group.

Anticancer effects of orally administered NACOS and COS have also been evaluated in MyD88 (myeloid differentiation primary response gene 88) knockout mice and were found to be related to MyD88-dependent as well as MyD88-independent pathways [27]. Stimulation of innate immunity is essential for activation of adaptive immunity [27] and in particular, Toll-like receptors (TLR) on the surface of intracellular organelles recognize specific structures on bacteria, viruses, and fungi [28]. In fact, chitin has been known to activate TLR-2 and Myd-88 in a novel IL-17A/IL-17AR-based innate immunity pathway [29] and adapter molecules such as MyD88 and Toll interleukin receptor (TIR)-domain-containing adapter-inducing interferon-β (TRIF) play important roles in inducing the production of cytokines via TLRs [30,31]. TLR-4 is also a known stimulator of cytokine production via MyD88 as well as TRIF signaling pathways [31]. In our previous experiments, we have observed that suppression of tumor growth following NACOS and COS treatments, administered orally, was not as robust in MyD88 knockout mice as it was in normal mice. These results suggest that *in vivo* antitumor effects of NACOS and COS are mediated not only by MyD88 dependent pathways, but also by MyD88 independent pathways.

Kan investigated the therapeutic effect of NACOS, administered through orally route, in patients with cancer [32]. A substantial regression of the cancer was observed in most patients, especially in those with early stage cancer. In addition, patients who were concomitantly treated with chemotherapy and/or surgical operation also showed significant decrease in tumor burden. The anticancer effects observed were regardless of the organ treated. These data reveal a potential for orally administered NACOS to be used in anti-cancer therapy. However, further detailed studies are required in order to successfully evaluate this.

## 4. Anti-Inflammatory Activities of COS

Numerous studies have reported the anti-inflammatory properties of COS. In a study conducted by Yoon *et al.* to investigate the effect of COS on LPS-stimulated RAW 264.7 cells, the researchers discovered that COS exposure led to a dose-dependent attenuation of LPS-induced secretion of TNF-α and IL-6 in the incubation medium [33]. Moreover, a corresponding decrease in TNF-alpha and IL-6 at the mRNA level indicated that COS exposure downregulated the expression of these cytokines at the transcription level. COS exposure was also found to decrease the lipopolysaccharide (LPS)-induced secretion of nitric oxide (NO) in the medium. Interestingly, the addition of external TNF-α to the medium reversed the COS-mediated decrease in IL-6 and NO levels thereby indicating that the anti-inflammatory effect of COS was by modulation of TNF-α pathway Yoon *et al.* have also investigated the protective effects of COS against glycerol-induced acute renal failure (a model of renal oxidative stress) [34] and their data indicate that COS mitigates the glycerol-induced inflammatory response, improves renal function, and has antioxidant effects in kidney. Fernandes *et al.* have demonstrated that the anti-inflammatory activity of COS in carrageenan-induced paw edema method was not only dose-dependent but also molecular weight-dependent at higher doses [35]. Quia *et al.* reported on the protective effect of COS in LPS-induced sepsis [36]. They found that treatment by COS not only attenuated organ dysfunction but also improved survival rate after LPS injection. To further dissect the mechanism, they examined several pro-inflammatory markers, including neutrophil infiltration in organs and TNF-α and IL-1β in serum, and found levels of these cytokines were significantly reduced in COS-treated animals. The redox imbalance in LPS-induced sepsis resulting from depletion of glutathione (GSH) and catalase (CAT) levels and increase in malondialdehyde (MDA) levels was also found to have been reversed by COS exposure. Furthermore, signal pathways activated by LPS, such as c-Jun NH(2)-terminal kinase (JNK) and p38 mitogen-activated protein kinase (MAPK), were also found to have been attenuated by COS treatment. These data demonstrate that the protection afforded by COS against LPS challenge in the mouse model could be by virtue of its anti-inflammatory as well as antioxidant properties.

Pangestuti *et al.* have described the effects of COS in four different molecular weight ranges (<1, 1–3, 3–5, and 5–10 kDa) for their ability to modulate inflammatory mediators in LPS-stimulated BV2 microglial cells [37]. At a concentration of 500 μg/mL, COS was found to attenuate the production of NO and prostaglandin E_2_ (PGE_2_) by inhibiting iNOS and COX-2 expression. Furthermore, the expression levels and release of inflammatory cytokines, including TNF-α, IL-6 andIL-1β, were also attenuated by COS. Notably, the inhibitory activity of COS was found to be dependent on its molecular weight, and lower molecular weight COS showed higher activity. In addition, this study confirmed the suppressive effects of COS on phosphorylation of JNK and p38 MAPK. Chung *et al.* have investigated the effects of COS against allergic reaction and allergy-induced asthma *in vivo* and *in vitro* [38]. COS, consisting of glucosamine (GlcN)(*n*), *n* = 3–5, was shown to be capable of inhibiting antigen-stimulated degranulation and cytokine generation in rat basophilic leukemia RBL-2H3 cells. This study also examined a protective effect of COS against ovalbumin (OVA)-induced lung inflammation in mouse model of asthma. The researchers discovered that animals receiving a daily oral administration of COS (16 mg/kg body weight/day) had a significant reduction in the mRNA expression and protein levels of IL-4, IL-5, IL-13, and TNF-α in their lung tissue and bronchoalveolar lavage fluid (BALF); protein levels of IL-4, IL-13, and TNF-α in BALF were decreased by 5.8-fold, 3.0-fold, and 9.9-fold, respectively, in comparison with the OVA-sensitized/challenged asthma control group. Choi *et al.* have demonstrated the effect of COS on body weight gain, adipocyte size, adipokine level, lipid profile, and adipose tissue gene expression profile in high-fat (HF) diet-induced obese mice [39]. Compared with the HF diet mice, mice fed HF diet supplemented with 3% COS had gained 15% less weight but did not display any change in food and energy intake. COS supplementation was also observed to have markedly improved the serum and hepatic lipid profiles. Microarray analysis revealed that dietary COS supplementation modulated adipogenesis-related genes such as matrix metallopeptidases 3, 12, 13, and 14, tissue inhibitor of metalloproteinase 1, and cathepsin K in the adipose tissues. Twenty-five percent of the COS-responsive genes identified are also involved in immune response, including inflammatory response and cytokine production. In a study conducted by Wei *et al.*, it was discovered that pretreatment with COS at 50–200 µg/mL could substantially abrogate NO production through the reduction of iNOS expression in LPS-activated L9 microglial cells [40]. In addition, COS was found to markedly decrease LPS-induced phosphorylation of p38 MAPK and extracellular signal-related protein kinase ½ and could also inhibit activation of NF-κB and activator protein-1 (AP-1) In a rat model of autoimmune anterior uveitis, Fang *et al.* discovered that COS treatment markedly attenuated the clinical scores and infiltration of leukocytes in the iris/ciliary body (ICB) in a dose-dependent manner [41]. The expression of inflammatory mediators such as TNF-α, iNOS, MCP-1 (Monocyte Chemotactic Protein-1), RANTES (CCL-5; regulated on activation normal T cell expressed and secreted), fractalkine, and intercellular adhesion molecule (ICAM)-1 was also substantially decreased in animals treated with COS. Moreover, in the ICB, COS decreased the degradation of IKB and levels of p65 thereby resulting in inhibition of DNA-binding by NF-KB. Under *in vitro* conditions, sensitized lymphocytes derived from the spleens of COS-treated animals had a reduced chemotactic mobility towards the aqueous humor and the levels of the previously mentioned inflammatory mediators in culture media was found to be reduced.

Li *et al.* have reported a mechanism by which COS attenuates inflammation in endothelial cells [42]. Regardless of the endothelial cell type, COS was found to be instrumental in suppressing the LPS-induced nuclear factor kappa-light-chain-enhancer of activated B cell (NF-κB)-dependent inflammatory gene expression, and this was associated with reduced nuclear translocation of NF-κB. LPS enhances O-GlcNAc modification of NF-κB/p65 and activates NF-κB pathway, and this could be prevented either by siRNA knockdown of O-GlcNAc transferase (OGT) or pretreatment with COS. Inhibition of MAPK or superoxide generation is also known to abolish LPS-induced NF-κB O-GlcNAcylation. Consistent with these observations, aortic tissue from LPS-treated mice showed enhanced NF-κB/p65 O-GlcNAcylation, and this was absent in tissues from mice that were pretreated with COS. Hence, COS-mediated attenuation of inflammatory response in vascular endothelial cells is most likely through decreased OGT-dependent O-GlcNAcylation of NF-κB. In a separate report, Li *et al.* stated that in porcine iliac artery endothelial cells (PIECs) treated with COS, the LPS-induced mRNA expression of E-selectin and ICAM-1 was reduced through the inhibition of p38 MAPK/ERK1/2 and NF-κB signal cascade. Inhibition of p38 MAPK and ERK1/2, also resulted in suppression of LPS-induced nuclear translocation of NF-κB p65. Both these effects were dose-dependent and ultimately inhibited adhesion of U973 cells to PIECs. Based on these results, it can be concluded that inhibition of MAPK phosphorylation and NF-κB activation in LPS-treated PIECs by COS results in decrease in expression of E-selectin and ICAM-1. Table 3 is a summary of the literature on these studies.

**Table 3:** A summary of anti-inflammatory activities of COS.

Cells or Model	Major Results	Ref.
RAW 264.7 cells (*in vitro*)	Exposured LPS-induced secration of TNF-α and IL-6; Decreased the LPS-induced secretion of NO	[33]
Acute renal failure model (*in vivo*)	Improved renal function and had antioxidant effects	[34]
Paw edema model (*in vivo*)	Sowed the anti-inflammatory effects according to the dose and MW dependent manner	[35]
Spesis model (*in vivo*)	Attenuated organ dysfunction and improved survival rate	[36]
BV2 microglial cells (*in vitro*)	Attenuated the production of NO and PGE_2_ by inhibiting iNOS and COX-2 expression; Decreased the expression levels of TNF-α, IL-6 and IL-1β. Suppressed the phosphorylations of JNK and p38MAPK	[37]
Asthma model (*in vivo*)	Reduced the mRNA expression and protein levels of IL-4, IL-5, IL-13 and TNF-α	[38]
Obese model (*in vivo*)	Reduced the weight gain by involving inflammatory response	[39]
L9 microglial cells (*in vitro*)	Abrogated NO production. Decreased the phosphorylation of p38 MAPK and inhibited activations of NF-κB and AP-1	[40]
Autoimmune anterior uveitis model (*in vitro*)	Attenuated the clinical score; Decreased the inflammation mediators such as TNF-α, iNOS, MCP-1, RANTES	[41]
Endothelial cells (*in vitro*)	Suppressed the activation of NF-κB pathways	[42]
Endothelial cells (*in vitro*)	Reduced mRNA expression of E-selectin and ICAM-1 through the inhibition of p38 MAPK/ERK1/2 and NF-κB cascade	[43]

## 5. Anti-Inflammatory Effects of COS for Inflammatory Bowel Disease

Inflammatory bowel disease (IBD) includes ulcerative colitis (UC), and Crohn’s disease, and is characterized by chronic inflammation of the gut [44]. Over the past 40 years, the incidence of IBD has steadily increased in some areas of the world [45], possibly due to changes in dietary habits (particularly consumption of diets low in fiber content) in these regions [46]. Yousef *et al.* have reported that in human colonic epithelial cell line, T84, subjected to LPS or TNF-α-stimulation, COS treatment prevented LPS binding to cells, NF-κB activation, production of TNF-α and IL-6, loss of epithelial barrier integrity, and TNF-α and oxidative stress-induced apoptosis [47]. They also discovered that in a mouse model of acute colitis, oral administration of COS protected against mortality and intestinal inflammation. In addition, NF-κB activation, and levels of TNF-α and IL-6 in colonic tissues were suppressed in mice that received COS. Importantly, COS administration after colitis induction was effective in ameliorating intestinal inflammation in acute [induced by 5% dextran sulfate sodium (DSS)] as well as chronic (induced by cyclic administration of 2.5% DSS) colitis models. These results suggest that COS may be effective in the treatment of IBD through inhibition of NF-κB signaling and apoptosis of intestinal epithelial cells.

**Figure 2:**
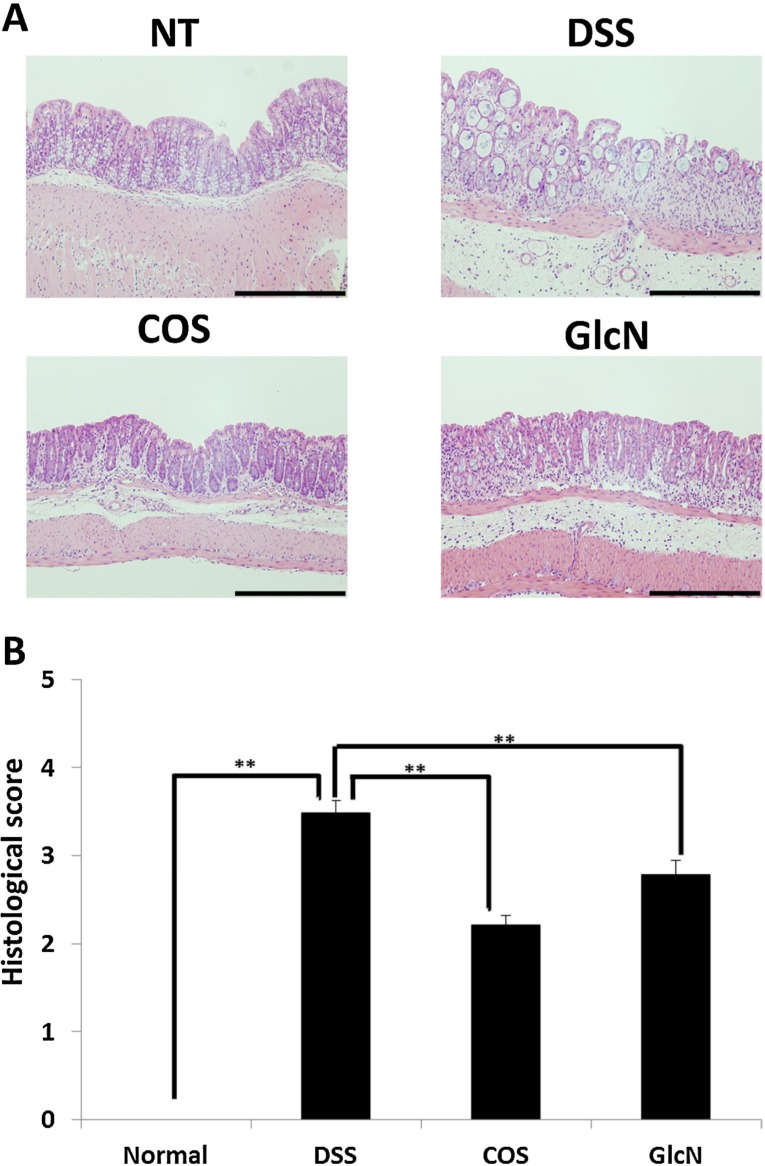
Effect of orally administered COS on colon injury in experimental IBD model. (**A**) Sections of colon tissue were stained with hematoxylin and eosin. Data are for one mouse per group from the NT, DSS, COS, and GlcN groups. Bar = 200 μm. (**B**) Data are the mean ± S.E. of 30 fields/100× magnification field in each group (Steel-Dwass test). ** *p* < 0.01. Reprinted with permission. Copyright 2015 Elsevier [48].

**Figure 3:**
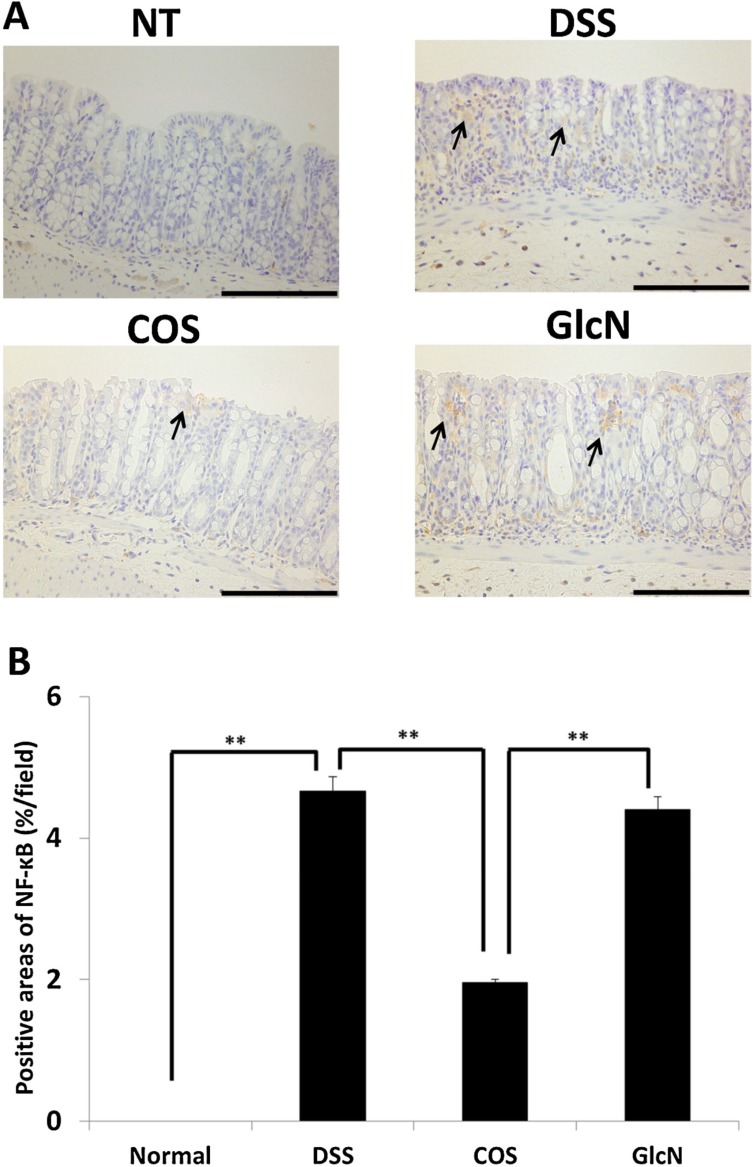
Effects of orally administered COS on NF-κB activation in colon in an experimental IBD model. (**A**) Areas stained positive for NF-κB are shown by arrows. Data are for one mouse per group from NT, DSS, COS, and GlcN groups. Bar = 100 μm. (**B**) Data are the mean ± S.E. of 30 fields/100× magnification field in each group (Steel-Dwass test). ** *p* < 0.01. Reprinted with permission. Copyright 2015 Elsevier [48].

**Figure 4:**
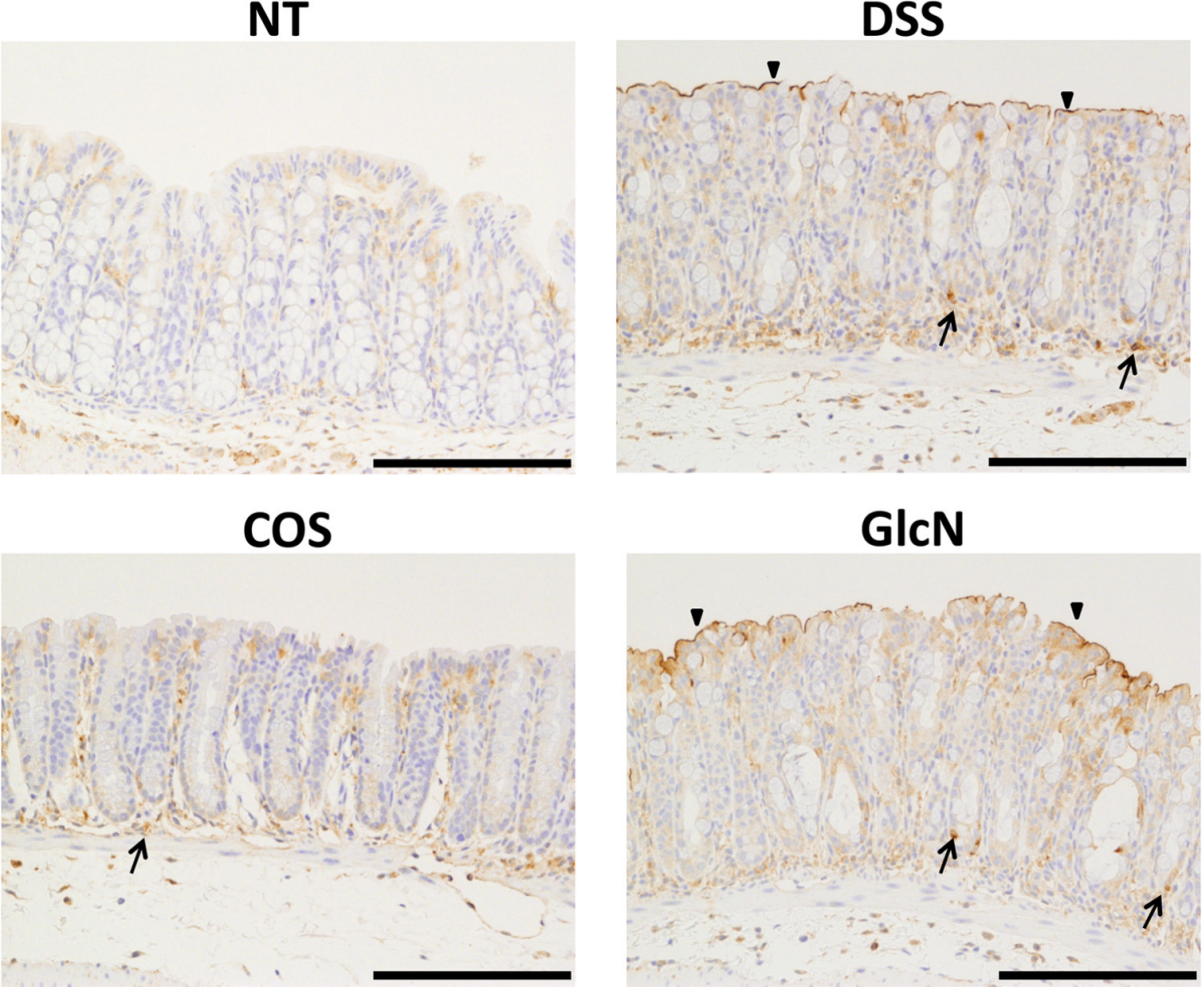
Effects of orally administered COS on iNOS activation in colon in an experimental IBD model. The immunohistochemistry of iNOS in the colon is shown. Areas stained positive for iNOS are shown by arrows and arrowheads. Data are for one mouse per group from NT, DSS, COS and GlcN groups. Bar = 100 μm. Reprinted with permission. Copyright 2015 Elsevier [48].

Our group has also evaluated the anti-inflammatory effects of orally administered COS in a mouse model [48] and discovered that COS improved shortening of colon length and tissue injury (as assessed by histology) (Figure 2). In addition, COS inhibited myeloperoxidase activation in inflammatory cells as well as activation of NF-κB, COX-2, and iNOS thereby preventing inflammation of colonic mucosa (Figure 3 and Figure 4).

NF-κB occupies a pivotal position in several signaling pathways involved in innate immunity. It stimulates expression of COX-2, PGE_2_, and pro-inflammatory cytokines (IL-6, TNF-α, and MCP-1) [49] and is the critical transcription factor needed to express genes associated with pro-inflammatory responses [50]. Cyclooxygenases are the enzymes responsible for biosynthesis of prostaglandins (from arachidonic acid) and these influence many biological processes, including homeostasis and inflammation [51]. In fact, COX-2 expression is increased mainly during inflammatory processes and cell transformation [52]. It has become increasingly clear that nitric oxide (NO) over-production by iNOS is deleterious to intestinal function [53,54], and iNOS levels are considered to be important determinants of colonic damage [55]. Hence, sustained overproduction of NO mediated by iNOS may have a role in the pathogenesis of IBD and induction of experimental colitis in the colon [54]. Oral administration of COS has been shown to reduce serum levels of pro-inflammatory cytokines (TNF-α and IL-6). Pro-inflammatory cytokines (IL-6, TNF-α, MCP-1) are known to trigger leukocyte activation and accumulation in tissues and play a significant role in inflammatory conditions, such as [56].

Our results suggest that a possible mechanism for the anti-inflammatory effects of orally administered COS is by suppression of inflammatory processes, including expression of NF-κB, COX-2, iNOS, and pro-inflammatory cytokines. Moreover, COS was found to prolong survival time in mice in an experimental model.

## 6. Next Step to Use NACOS, COS and Its Derivatives for Patient

**Figure 5:**
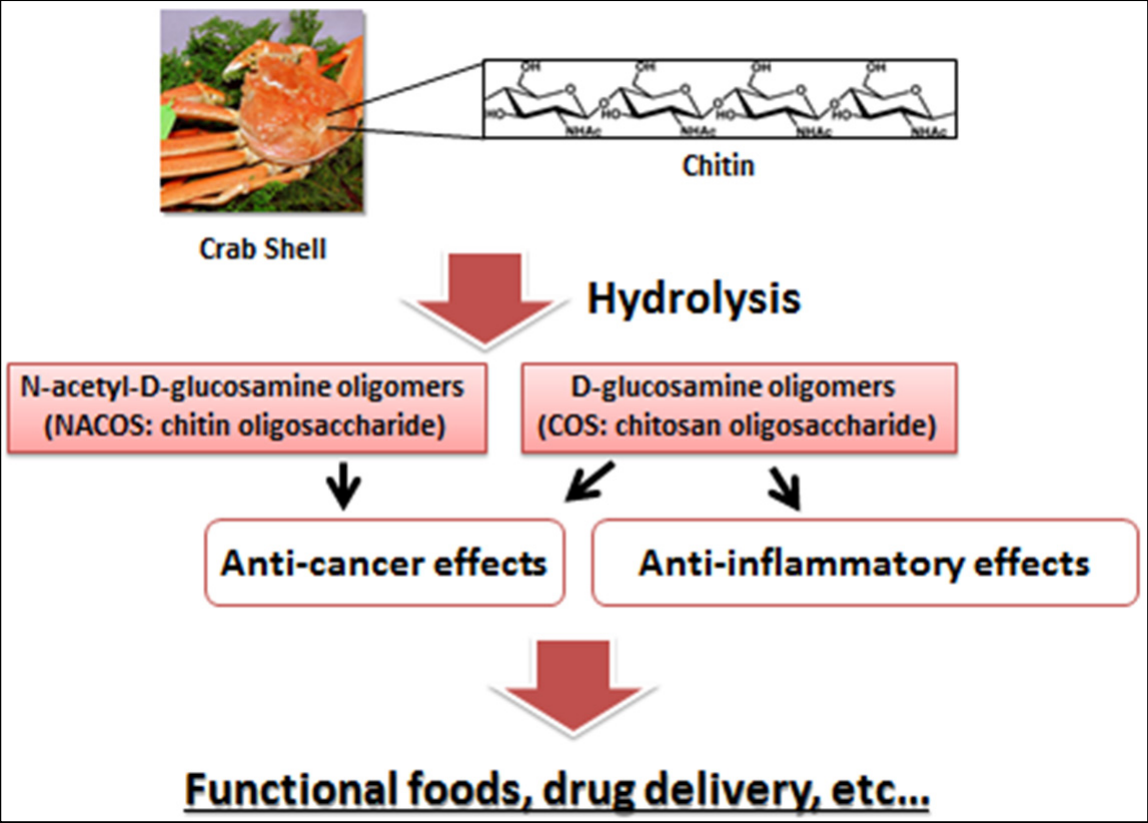
A schema of this review.

A schema of this review is shown in Figure 5. An irrefutable amount of evidence has already established the anticancer and anti-inflammatory properties of NACOS and COS in experimental models. More recently, it has been shown that the beneficial traits are retained when NACOS and COS are administered by the oral route. To our knowledge, one article reported the safety of oral administration of COS by short-term study [57].

However, the exact mechanisms behind the actions of NACOS and COS are not yet fully dissected, and further mechanistic studies will be required to harness the benefits of NACOS and COS in therapeutics. More recently, beneficial effects of nanomaterials based on chitin and chitosan are also reporting [2,58,59,60,61,62,63]. Effective usage including combination of nanomaterials from chitin and chitosan with NACOS and COS is must be researched.
